# Supplementary material for: An ubiquitin-dependent balance between mitofusin turnover and fatty acids desaturation regulates mitochondrial fusion
Source: Nat Commun. 2017 Jun 13;8:15832. doi: 10.1038/ncomms15832 (PMC5474747; doi:10.1038/ncomms15832)
Supplement: Supplementary Information — Supplementary Figures, Supplementary Tables, and Supplementary References [file ncomms15832-s1.pdf]

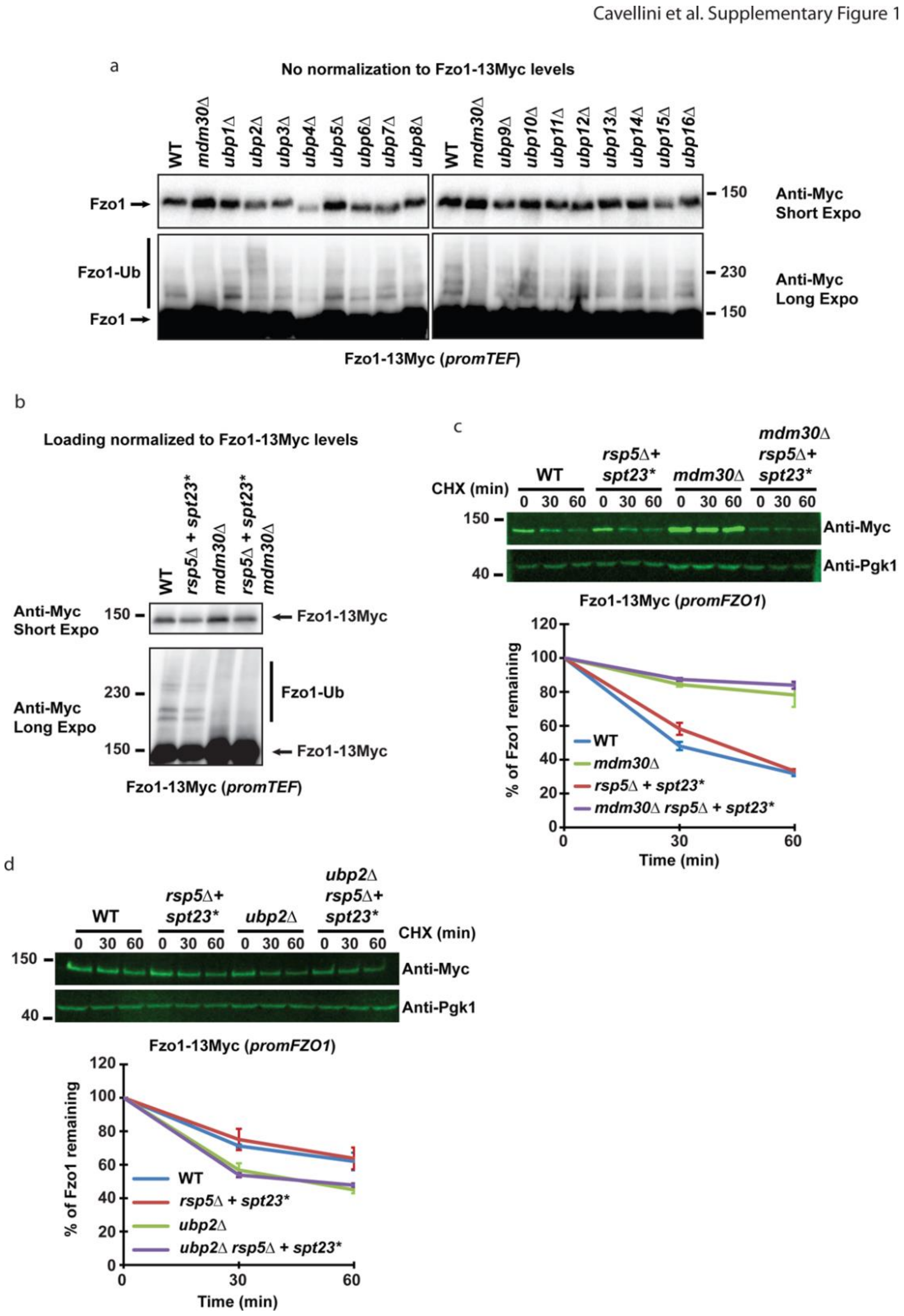


**Supplementary Figure 1: Fzo1 ubiquitylation and degradation do not depend on Rsp5 (related to Figure 1).**

**(a)** Fzo1 ubiquitylation in *UBP* mutants. Total protein extracts prepared from WT, *mdm30Δ* and *ubpXΔ* cells (BY4741 background) transformed with pRS416-TEF-FZO1-13MYC were analyzed by anti-Myc immunoblotting. Molecular weights (MW) in kDa are shown on the right of short or long exposures of indicated regions of anti-Myc immunoblots. The loading of the extracts was not normalized to Fzo1-13Myc levels. Note that in this context, the intensity of Fzo1-13Myc high MW species is proportional to the level of unmodified Fzo1-13Myc except in *mdm30Δ* and *ubp2Δ* cells where the pattern of high MW bands is respectively no longer detected or affected. **(b)** Fzo1 ubiquitylation by Mdm30 is not affected in cells lacking Rsp5. Total protein extracts prepared from WT, *rsp5Δ+spt23**, *mdm30Δ* and *rsp5Δ+spt23* mdm30Δ* cells (DF5 background) transformed with pRS414-TEF-FZO1-13MYC were analyzed by anti-Myc immunoblotting. Molecular weights (MW) in kDa are shown on the left of short or long exposures of indicated regions of anti-Myc immunoblots. Note the doublet characteristic of Fzo1 ubiquitylation that is detected in WT but not *mdm30Δ* cells. **(c)** Mdm30-mediated degradation of Fzo1 is not affected in cells lacking Rsp5. Total protein extracts from WT, *rsp5Δ+spt23**, *mdm30Δ* and *rsp5Δ+spt23* mdm30Δ* cells (DF5 background) transformed with pRS414-FZO1-13MYC were prepared at indicated time points after addition of CHX and analyzed by anti-Myc and anti-Pgk1 immunoblotting followed by detection with fluorescent secondary antibodies (Top). Quantification of CHX chases (Bottom) were performed by normalization of Fzo1 levels to those of Pgk1; error bars represent s.e.m. from three independent experiments. **(d)** Absence of Rsp5 does not affect the faster Fzo1 turnover seen in cells that lack Ubp2. Total protein extracts from WT, *rsp5Δ+spt23**, *ubp2Δ* and *rsp5Δ+spt23* ubp2Δ* cells (DF5 background) transformed with pRS414-FZO1-13MYC and grown at 30°C were prepared at indicated time points after addition of CHX and analyzed by anti-Myc and anti-Pgk1 immunoblotting followed by detection with fluorescent secondary antibodies (Top). Quantification of CHX chases (Bottom) were performed by normalization of Fzo1 levels to those of Pgk1; error bars represent the s.e.m. from three independent experiments.

Cavellini et al. Supplementary Figure 2

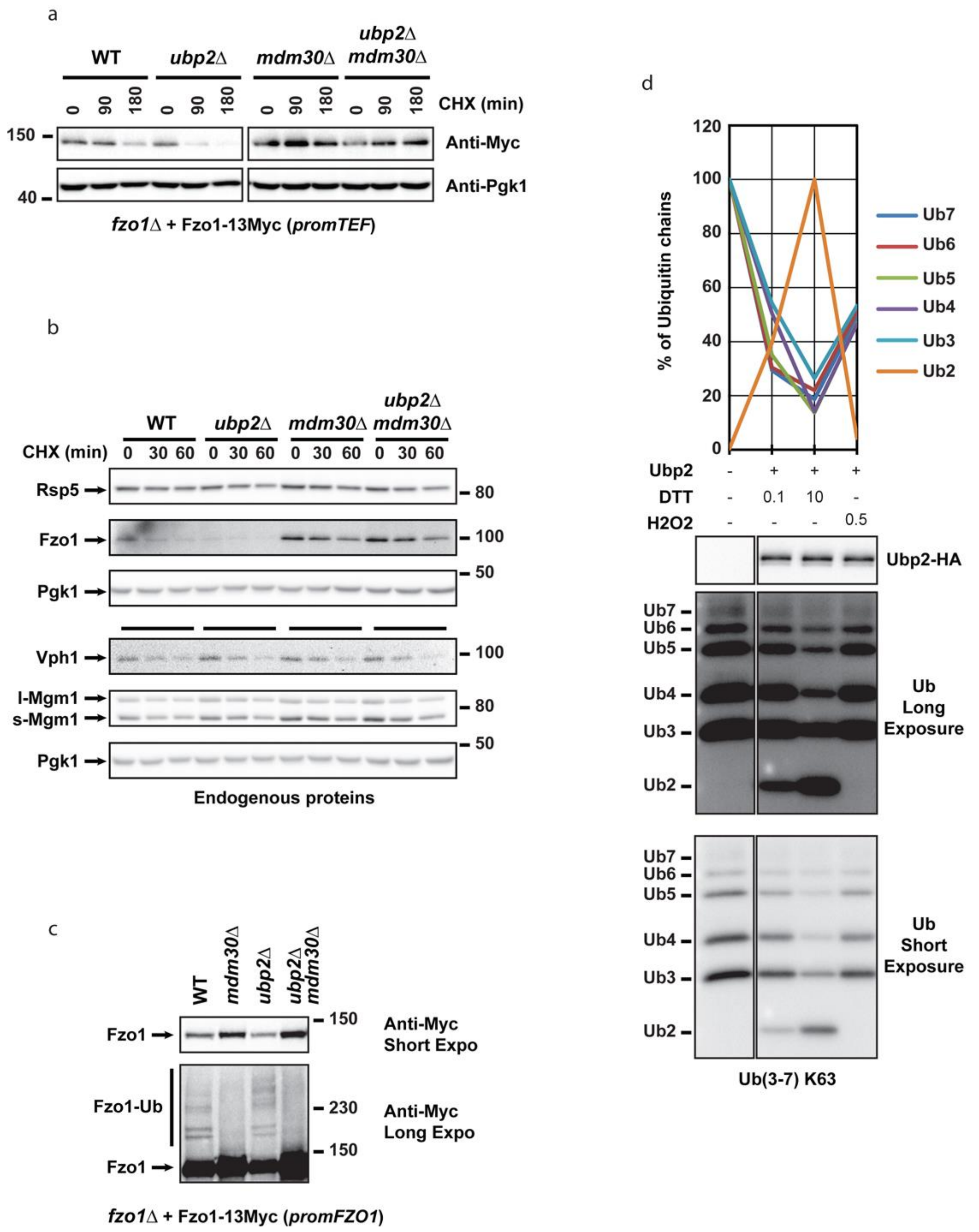


**Supplementary Figure 2: Mdm30 regulates the ubiquitylation and degradation of Fzo1 in cells that lack Ubp2 (related to Figure 2).**

**(a)** Degradation of overexpressed Fzo1 is accelerated in *ubp2Δ* but abolished in *ubp2Δ mdm30Δ* cells. CHX chases with *WT*, *mdm30Δ*, *ubp2Δ* and *ubp2Δ mdm30Δ* cells (MCY572 background) shuffled with pRS414-TEF-FZO1-13MYC were analyzed by anti-Myc and anti-Pgk1 immunoblotting. Molecular weights (MW) in kDa are shown on the left of immunoblots. **(b)** Degradation of endogenous Fzo1 is specifically accelerated in *ubp2Δ* but abolished in *ubp2Δ mdm30Δ* cells. CHX chases with *WT*, *mdm30Δ*, *ubp2Δ* and *ubp2Δ mdm30Δ* cells (W303 background) were analyzed by anti-Rsp5, anti-Fzo1, anti-Vph1, anti-Mgm1 and anti-Pgk1 immunoblotting. Molecular weights (MW) in kDa are shown on the right of immunoblots. Note that as compared to endogenous Fzo1, the stabilities of endogenous Rsp5, endogenous Vph1 and endogenous long and short forms of Mgm1 (l-Mgm1 and s-Mgm1) are not affected by the absence of Ubp2, Mdm30 or both. **(c)** Impact of Ubp2 on Mdm30-mediated ubiquitylation of Fzo1 expressed under physiologic conditions. Total protein extracts prepared from WT, *mdm30Δ*, *ubp2Δ* and *ubp2Δ mdm30Δ* cells (MCY572 background) shuffled with pRS414-FZO1-13MYC were analyzed by anti-Myc immunoblotting. Molecular weights (MW) in kDa are shown on the right of short or long exposures of indicated regions of anti-Myc immunoblots. Note the accumulation of Mdm30-dependent high MW species of Fzo1 migrating above 230kDa in *ubp2Δ* cells. **(d)** *In vitro* deubiquitylation assays. Ub(3-7)K63 chains were incubated with mock (-) or Ubp2-HA immunoprecipitates in the absence (-) or in the presence of DTT (0.1 or 10 mM) or H2O2 (0.5 mM). Reactions were analyzed by anti-HA and anti-Ub immunoblotting. Long (Center) and short (Bottom) exposures of Ub blots are shown. The level of each length of chain was quantified relative to the mock (- Ubp2) condition (Top). Note that in the presence of DTT, Ubp2 can disassemble all types of K63-linked chains regardless of their length to ultimately generate Ub2 K63-linked dimers.

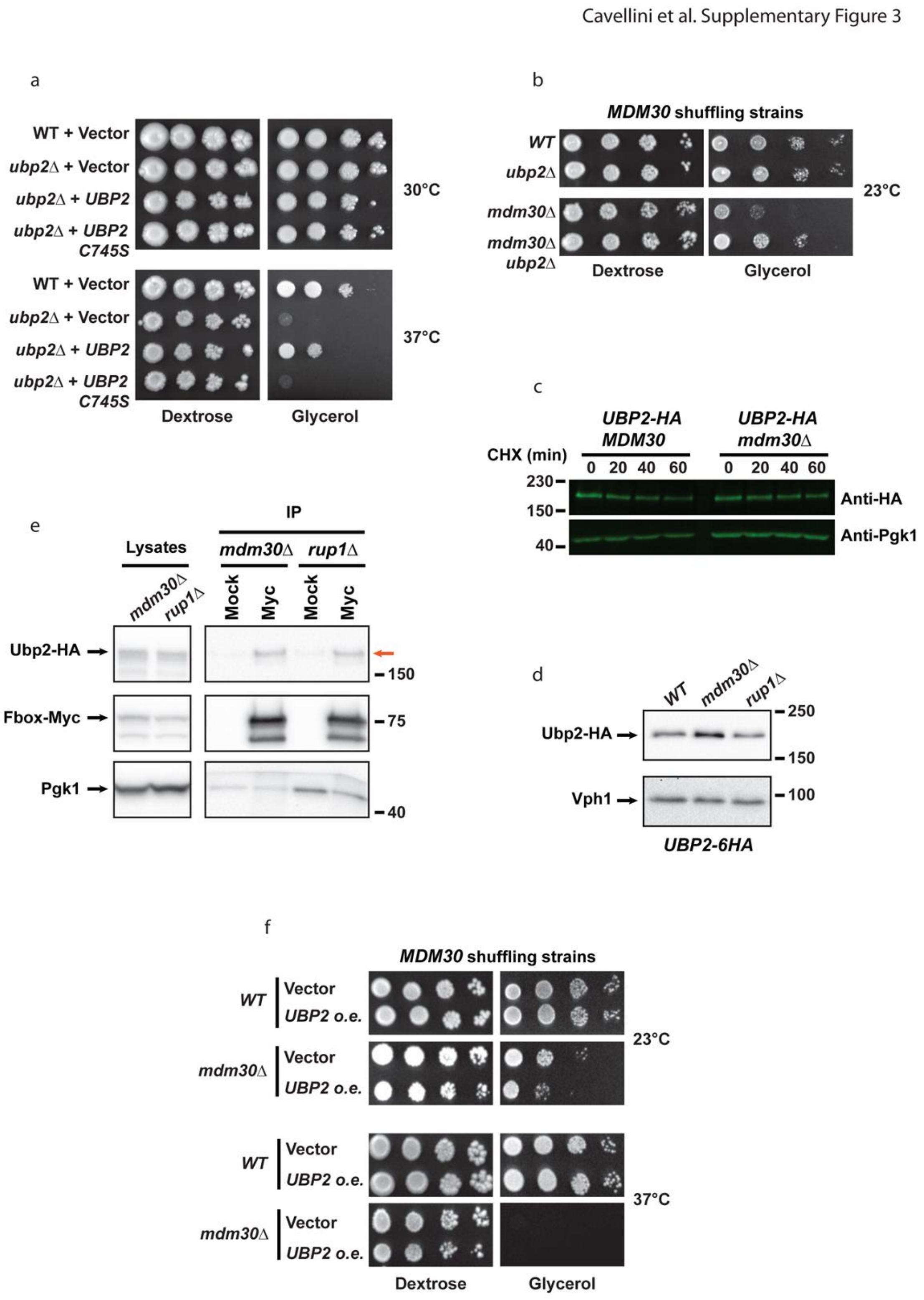


**Supplementary Figure 3: Ubp2 is regulated by Mdm30 and is causal in the respiration defect of *mdm30Δ* cells (related to Figure 3).**

**(a)** The respiratory growth defect of *ubp2Δ* cells at 37°C. Serial dilutions of *WT* (MCY554) and *ubp2Δ* (MCY1251) strains transformed with an empty plasmid (Vector), a plasmid expressing wild-type Ubp2 (*UBP2*) or a plasmid expressing a catalytic mutant of Ubp2 (*UBP2 C745S*) were grown in the presence of glucose or glycerol as the sole carbon source at 30 or 37°C. Note the specific growth defect of cells lacking functional Ubp2 at 37°C. **(b)** Dextrose and glycerol spot assays from Fig. 3a and 3b at 23°C. Serial dilutions of *MDM30* (MCY971) and *MDM30 ubp2Δ* (MCY996) shuffling strains were grown in the presence of glucose or glycerol as the sole carbon source at 23°C, before (*WT* and *ubp2Δ*) or after (*mdm30Δ* and *mdm30Δ ubp2Δ*) cure of the *MDM30* shuffle plasmid. Note the improved growth on glycerol media at 23°C of *mdm30Δ ubp2Δ* cells as compared to *mdm30Δ* cells. This result suggests that Ubp2 contributes to mitochondrial fusion deficiency in *mdm30Δ* cells. **(c)** Example of CHX chase from Fig. 3f. Total protein extracts from *UBP2-6HA* (MCY968) and *UBP2-6HA mdm30Δ* (MCY1031) strains were prepared at indicated time points after addition of CHX and analyzed by anti-Myc and anti-Pgk1 immunoblotting followed by detection with fluorescent secondary antibodies. **(d)** Rup1 is not required for Ubp2 degradation. Total protein extracts from *UBP2-6HA* (MCY968), *UBP2-6HA mdm30Δ* (MCY1031) and *UBP2-6HA rup1Δ* (MCY1031) strains grown in YPD were analyzed by anti-HA and anti-Vph1 immunoblotting. Note that the level of Ubp2-HA is similar in WT and *rup1Δ* cells but is increased upon deletion of *MDM30* **(e)** Rup1 is not required for binding between Ubp2 and the F-box mutant of Mdm30. *ubp2*Δ *mdm30*Δ (*mdm30*Δ) and *ubp2*Δ *rup1*Δ (*rup1*Δ) cells co-expressing Ubp2-HA and the F-box mutant of Mdm30-Myc (Fbox-Myc) were lysed and lysates were subjected to co-IP with anti-Myc or Mock antibody followed by immunoblotting as indicated. Left panels, lysates (10% input of IP); right panels, immunoprecipitates. Red arrow indicates co-IP between Ubp2-HA and the f-box mutant of Mdm30-Myc. Pgk1 was used as a loading and IP control. **(f)** Dextrose and glycerol spot assays from Fig. 3g at 23 and 37°C. Serial dilutions of *MDM30* shuffling strains (MCY971) transformed with pRS423-UBP2-6HA (*UBP2 o.e.*) or an empty vector (pRS423) were grown in the presence of glucose or glycerol as the sole carbon source at 23 and 37°C, before (*MDM30*) or after (*mdm30Δ*) cure of the *MDM30* shuffle plasmid. Note the delayed growth on glycerol media at 23°C of *mdm30Δ* cells overexpressing Ubp2 as compared to those transformed with the empty vector.

Cavellini et al. Supplementary Figure 4

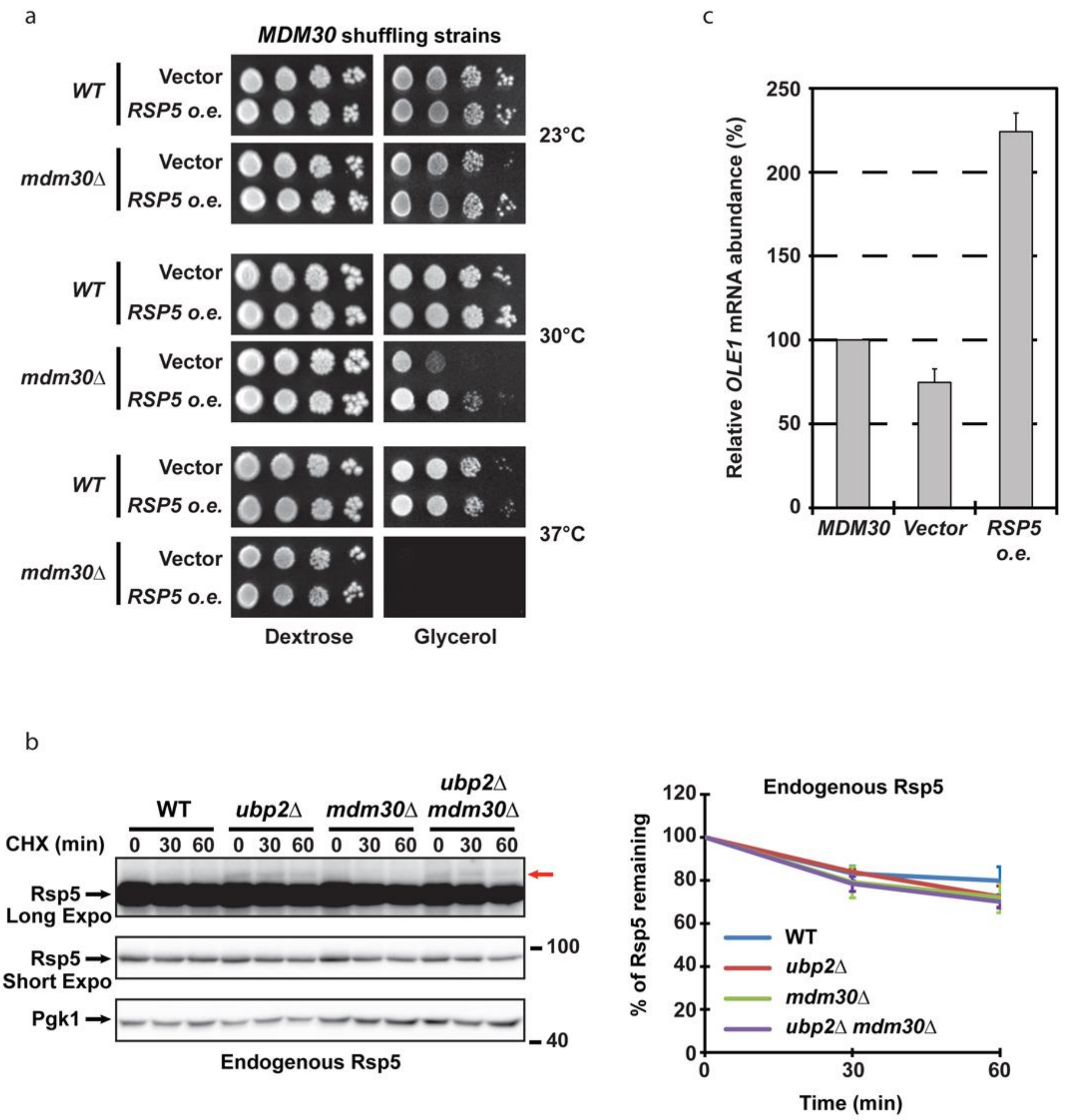


**Supplementary Figure 4: The Rsp5-dependent *OLE1*-pathway is down-regulated in *mdm30Δ* cells (related to Figure 4).**

**(a)** Dextrose and glycerol spot assays from Fig. 4b at 23, 30 and 37°C. Serial dilutions of *MDM30* shuffling strains (MCY971) transformed with pRS315-RSP5 or an empty vector (pRS425) were grown in the presence of glucose or glycerol as the sole carbon source at 23, 30 and 37°C, before (*WT*) or after (*mdm30Δ*) cure of the *MDM30* shuffle plasmid. **(b)** Endogenous

Rsp5 levels and turnover are not affected in cells lacking Ubp2, Mdm30 or both. Total protein extracts from WT, *ubp2Δ*, *mdm30Δ* and *ubp2Δ mdm30Δ* cells (W303 background) were prepared at indicated time points after addition of CHX and analyzed by anti-Rsp5 and anti-Pgk1 immunoblotting. MW in kDa are shown on the right of short or long exposures of indicated regions of immunoblots (Left). Quantification of CHX chases (Right) were performed by normalization of Rsp5 levels to those of Pgk1; error bars represent s.e.m. from three independent experiments. Note the auto-ubiquitylation species of Rsp5 (red arrow) that accumulate in the absence of Ubp2. **(c)** *OLE1* mRNAs abundance in *mdm30Δ* cells. Relative *OLE1* mRNA levels in *mdm30Δ* strains (MCY971) shuffled with *MDM30*, *RSP5* or empty plasmids were determined by qPCR. The *OLE1* mRNA abundance in *RSP5* and empty vectors conditions was normalized to *ACT1* mRNA levels and expressed relative to *OLE1* expression in *MDM30* positive cells using the Livak method. Error bars represent the s.d from six reactions. Note the ~25% decrease and the more than two-fold increase in *OLE1* transcripts from vector and *RSP5* conditions, respectively.

Cavellini et al. Supplementary Figure 5

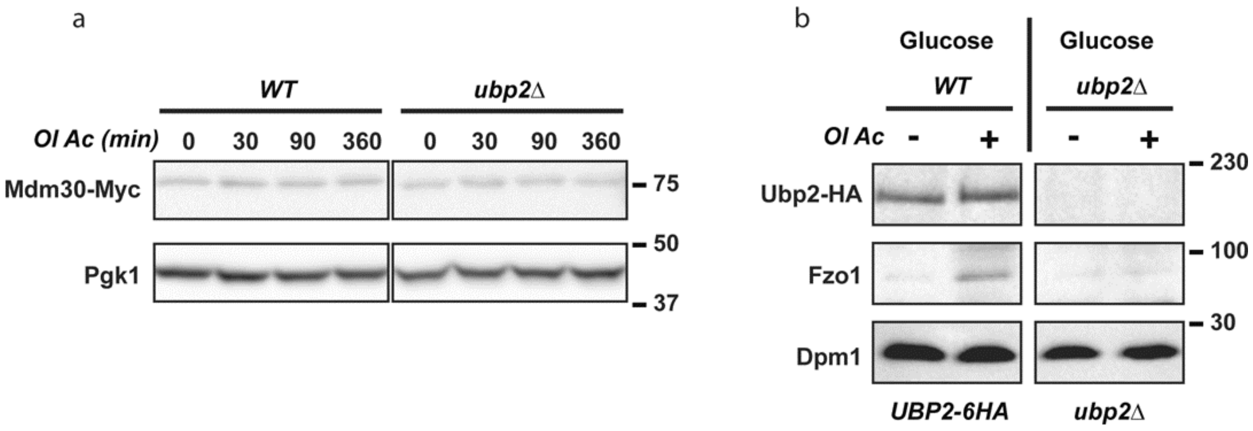


**Supplementary Figure 5: Mdm30 levels and activity are not affected after treatment with UFAs. (related to Figure 5b).**

**(a)** Mdm30 levels do not significantly vary upon treatment with UFAs. *ubp2*Δ *mdm30*Δ cells co-expressing Ubp2-HA and Mdm30-Myc (*WT*) or expressing Mdm30-Myc only (*ubp2*Δ) were grown for 360 minutes in the absence (0) or in the presence of 0.1% Oleic Acid added 30, 90 or 360 minutes prior preparation of total protein extracts. Resulting extracts were analyzed by anti-Myc and anti-Pgk1 immunoblotting. **(b)** Upregulation of Fzo1 levels after treatment with UFAs is abolished upon deletion of *UBP2*. Total protein extracts from *UBP2-6HA* (MCY968) and *ubp2Δ* (MCY1251) strains grown in YPD (Glucose) in the absence (-) or in the presence (+) of 0.1% Oleic Acid were analyzed by anti-HA, anti-Fzo1 and anti-Dpm1 immunoblotting. Note that as opposed to WT cells, the levels of Fzo1 do not increase upon treatment with Oleic Acid when Ubp2 is not expressed. This demonstrates that stabilization of Fzo1 is caused by increased Ubp2-dependent deubiquitylation resulting from stabilization of the DUB after treatment with UFAs. MW in kDa are shown on the right of indicated regions of immunoblots in (a) and (b).

Cavellini et al. Supplementary Figure 6

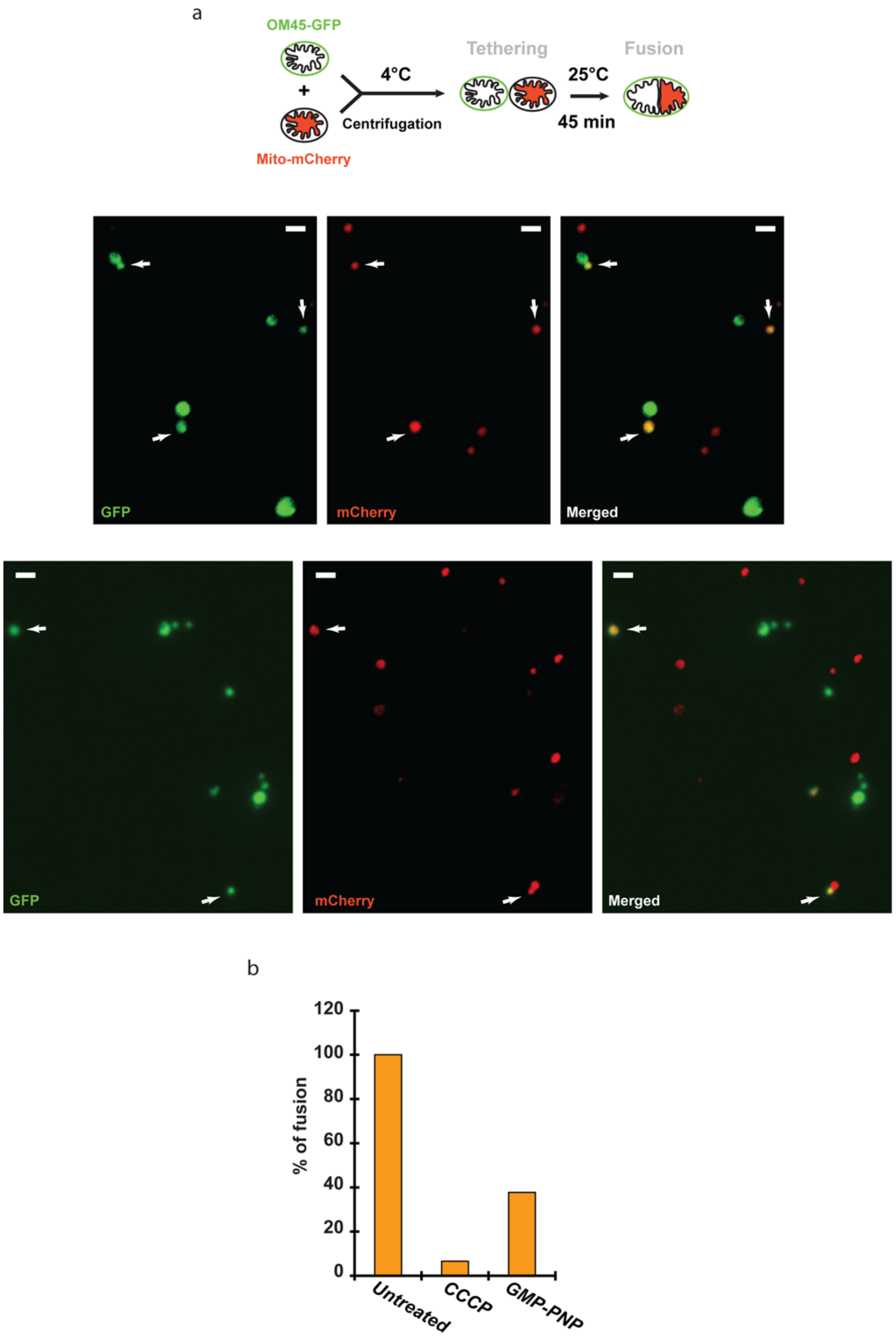


**Supplementary Figure 6: Fluorescence microscopy analysis of *in vitro* outer membrane fusion reactions. (related to Figure 5d).**

**(a)** *In vitro* outer membrane fusion reactions. Top: Fusion reactions were performed by mixing mitochondria isolated from cells expressing either the outer membrane protein OM45 tagged with GFP (OM45-GFP) or the mitochondrial matrix targeted mCherry (Mito-mCherry). Bottom: Co-localization of GFP and mCherry indicate intermediates with fused outer membranes (white arrows). Two distinct fields are shown. ; Scale bars 2 μM **(b)** *In vitro* fusion of outer membranes is inhibited by CCCP and GMP-PNP. Reactions were performed with mitochondria purified from DF5 OM45-GFP and DF5 Mito-mCherry cells either untreated or treated with 0.2 mM CCCP or 12 mM GMP-PNP. % of outer membrane fusion efficiency in the presence of CCCP or GMP-PNP was calculated relative to the untreated reaction.

Cavellini et al. Supplementary Figure 7

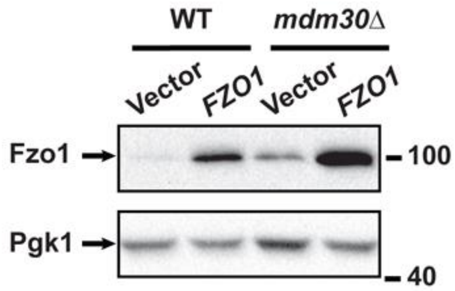


**Supplementary Figure 7: The *FZO1* extra copy induces higher levels of mitofusin in *WT* and *mdm30Δ* cells (related to Figure 6).**

Total protein extracts of *MDM30* (MCY971) shuffle strains either covered by (WT) or cured from (*mdm30Δ*) the *MDM30* shuffle plasmid and transformed with pRS314-*FZO1* or an empty vector (pRS314) were analyzed by anti-Fzo1 and anti-Pgk1 immunoblotting. Note that the extra FZO1 copy increases Fzo1 levels in both WT and *mdm30Δ* cells.

Cavellini et al. Supplementary Figure 8

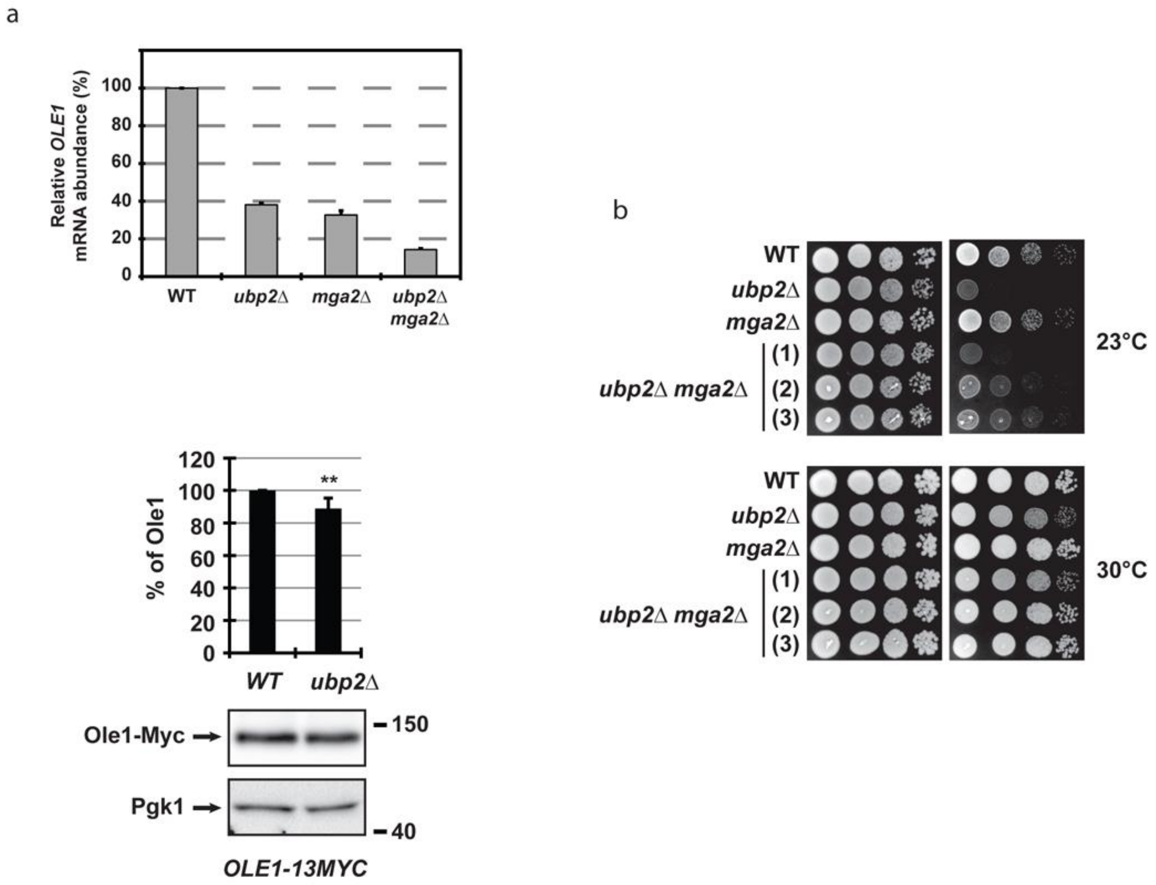


**Supplementary Figure 8: Low levels of Fzo1 and UFAs support partial restoration of respiratory growth in cells lacking Ubp2 (related to Figure 7).**

**(a)** Ole1 mRNA and protein levels in *ubp2Δ* and *mga2Δ* cells. Top: Relative *OLE1* mRNA levels in *WT*, *ubp2Δ*, *mga2Δ* and *ubp2Δ mga2Δ* cells (DF5 background) analyzed by qPCR. Error bars represent the s.d. from six reactions. Deletion of *MGA2* induced a 60% decrease in *OLE1* mRNAs, which is consistent with the 51% decrease of the endogenous Ole1 protein previously documented in *mga2Δ* cells [1]. In *ubp2Δ* cells, a similar 60% diminution in *OLE1* mRNAs was surprisingly observed. Bottom: Total protein extracts from *OLE1-13MYC* (MCY1126) and *OLE1-13MYC ubp2Δ* (MCY1032) strains were analyzed by anti-Myc and anti-Pgk1 immunoblotting; Ole1-Myc levels were quantified in *ubp2Δ* relative to *WT* cells. Error bars represent the s.d. from three independent experiments. **P < 0.05 (one-way analysis of variance (ANOVA)). The weak decrease of Ole1 protein levels in *ubp2Δ* cells (5 to 10%) contrasts with the 60% decrease of *OLE1* transcripts. This behavior of Ole1 expression in *ubp2Δ* cells is, at this

stage, not fully understood but may be linked to inhibitory Rsp5 auto-ubiquitylation in *UBP2* null cells ([2] and supplementary Fig. 4b; red arrow) combined with Mga2-mediated stabilization of diminished *OLE1* transcripts [3]. **(b)** Dextrose and glycerol spot assays from Fig. 7c at 23 and 30°C. Serial dilutions of WT (MCY554), *ubp2Δ* (MCY1147), *mga2Δ* (MCY1078) and *ubp2Δ mga2Δ* strains were grown in the presence of glucose or glycerol as the sole carbon source at 23, 30 and 37°C. Three distinct *ubp2Δ mga2Δ* clones issued from mating between *ubp2Δ* and *mga2Δ* strains are shown. All subsequent experiments were performed with clone 3 (MCY1098). Note that *ubp2Δ mga2Δ* clones 2 and 3 display improved growth as compared to *ubp2Δ* cells on glycerol media at 23°C.

Cavellini et al. Supplementary Figure 9

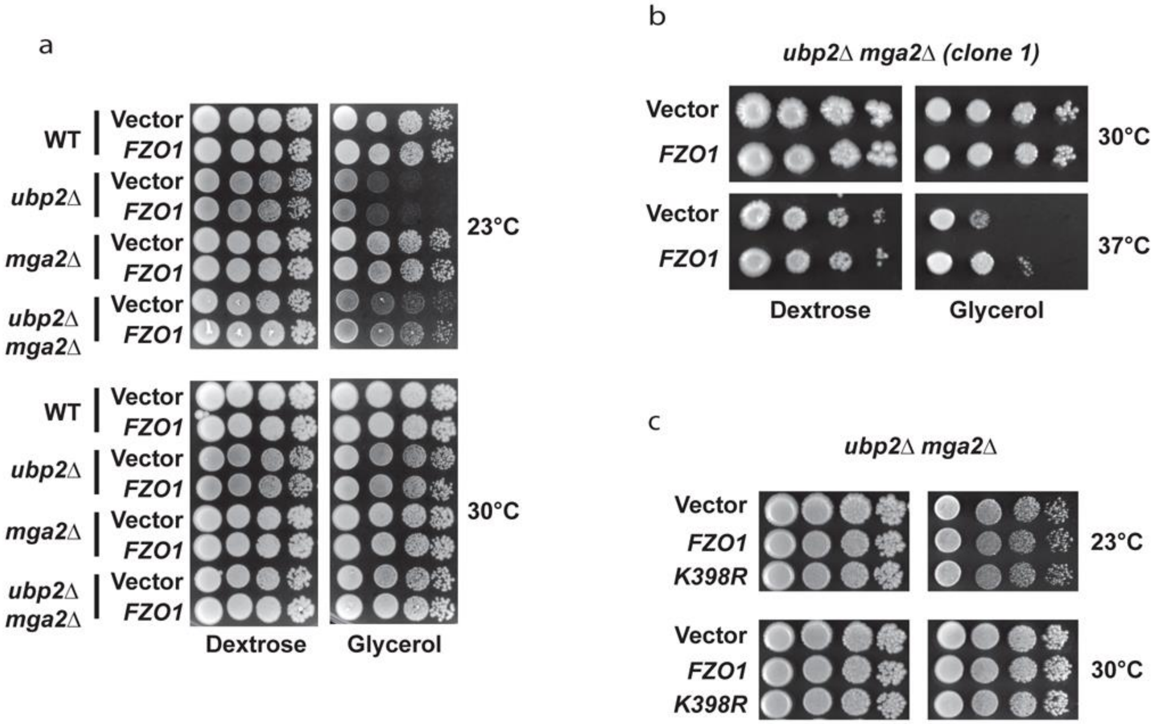


**Supplementary Figure 9: Impact of the *FZO1* extra copy in *ubp2∆ mga2∆* cells (related to Figure 8).**

**(a)** Dextrose and glycerol spot assays from Fig. 7b at 23 and 30°C. Serial dilutions of WT (MCY554), *ubp2∆* (MCY1147), *mga2∆* (MCY1078) and *ubp2∆ mga2∆* (MCY1098) strains transformed with an empty plasmid (Vector) or pRS314-FZO1 (*FZO1*) were grown in the presence of glucose or glycerol as the sole carbon source at 23 and 30°C. Note the improved respiratory growth of *ubp2∆ mga2∆* as compared to *ubp2∆* at 23°C. **(b)** Serial dilutions of the *ubp2∆ mga2∆* clone 1 strain transformed with an empty plasmid (Vector) or pRS314-FZO1 (*FZO1*). Cells were grown in the presence of glucose or glycerol as the sole carbon source at 30 and 37°C. Note the positive impact of the *FZO1* extra copy on the respiratory growth of this *ubp2∆ mga2∆* clone at 37°C for which respiratory growth was delayed as compared to clones 2 and 3 (Fig. 7c). This emphasizes the specificity of the *FZO1* extra-copy impact on *ubp2∆ mga2∆* strains **(c)**Serial dilutions of *ubp2∆ mga2∆* (MCY1098) strains transformed with an empty plasmid (Vector), pRS314-FZO1 (*FZO1*) or pRS314-FZO1 K398R (*K398R*) were grown in the presence of glucose or glycerol as the sole carbon source at 23 and 30°C (Related to Fig. 8e).

Cavellini et al. Supplementary Figure 10

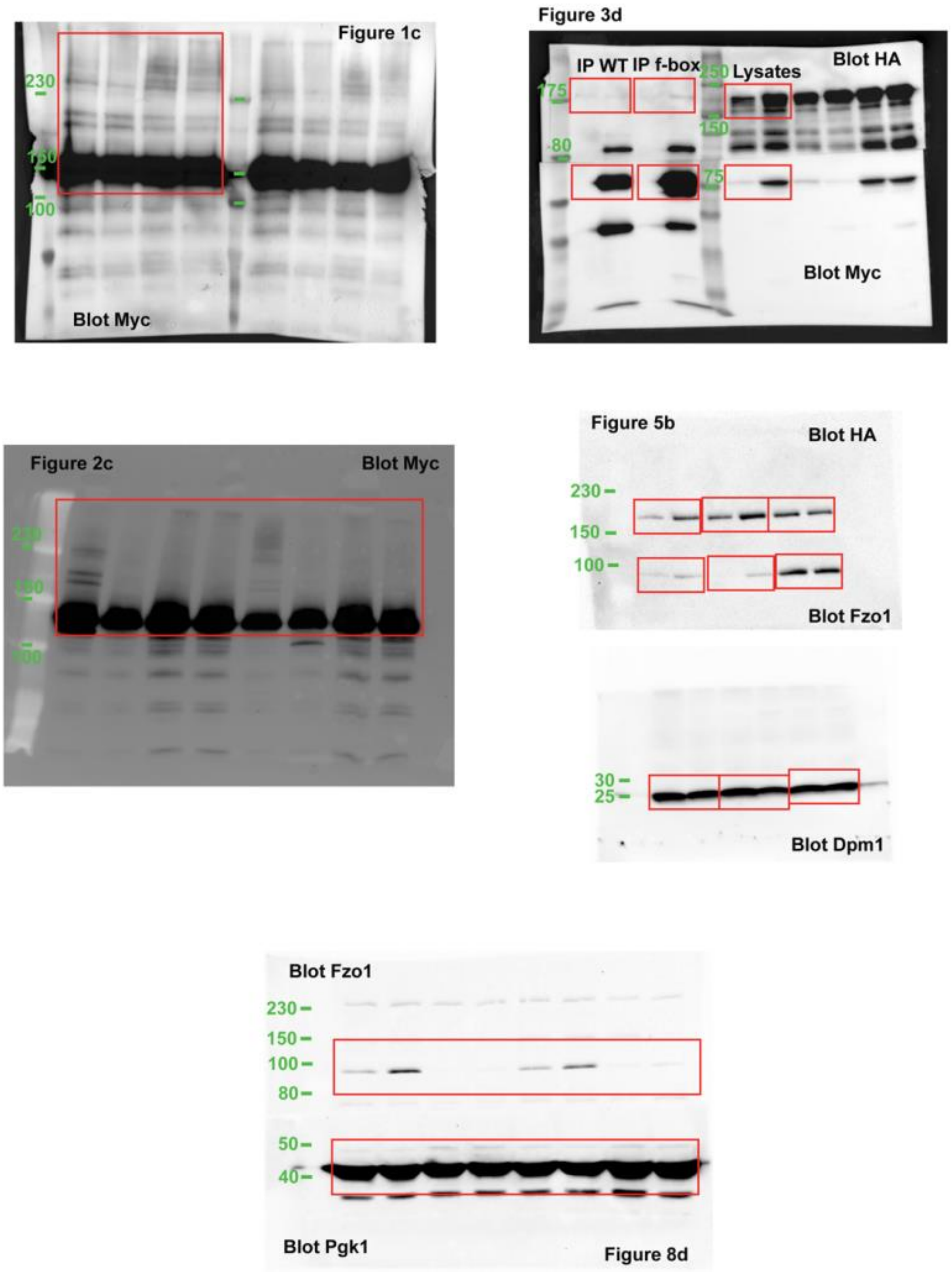


**Supplementary Figure 10: Uncropped scans of the most important blots**

## Supplementary Table 1: *S. cerevisiae* strains used in this study.

| Name | Parental strains | Genotype | Occurrence in the study | Reference |
|---|---|---|---|---|
| WT BY4741 (MCY338) | BY4741 | *Mata his3Δ1 leu2Δ0 met15Δ0 ura3Δ0* | 1b, 1e, 1f | Euroscarf |
| mdm30Δ (MCY352) | BY4741 | *Mata his3Δ1 leu2Δ0 met15Δ0 ura3Δ0 mdm30Δ::kanMX4* | 1b | Euroscarf |
| ubp2Δ (MCY866) | BY4741 | *Mata his3Δ1 leu2Δ0 met15Δ0 ura3Δ0 ubp2Δ::kanMX4* | 1b, 1e, 1f | Euroscarf |
| ubp12Δ (MCY876) | BY4741 | *Mata his3Δ1 leu2Δ0 met15Δ0 ura3Δ0 ubp12Δ::kanMX4* | 1b | Euroscarf |
| ubp10Δ (MCY874) | BY4741 | *Mata his3Δ1 leu2Δ0 met15Δ0 ura3Δ0 ubp10Δ::kanMX4* | 1b | Euroscarf |
| ubp15Δ (MCY879) | BY4741 | *Mata his3Δ1 leu2Δ0 met15Δ0 ura3Δ0 mdm15Δ::kanMX4* | 1b | Euroscarf |
| ubp16Δ (MCY880) | BY4741 | *Mata his3Δ1 leu2Δ0 met15Δ0 ura3Δ0 mdm16Δ::kanMX4* | 1b | Euroscarf |
| rup1Δ (MCY357) | BY4741 | *Mata his3Δ1 leu2Δ0 met15Δ0 ura3Δ0 rup1Δ::kanMX4* | 1e, 1f | Euroscarf |
| W303 WT (MCY553) | W303 | *Mata ura3-1 trp1-1 leu2-3,112 his3-11,15 can1-100* | Used to build FZO1 and MDM30 shuffle strains | Gift from T. Teixeira |
| W303 WT (MCY554) | W303 | *MATα ura3-1 trp1-1 leu2-3,112 his3-11,15 can1-100* | | Gift from T. Teixeira |
| *FZO1* (MCY572) | W303 | *MATα ura3-1 trp1-1 leu2-3,112 his3-11,15 can1-100 fzo1Δ::LEU2* pRS416-FZO1 | 2a-b, 2c, 5c, S2a-c | This study |
| *FZO1 mdm30Δ* (MCY585) | MCY572 | *MATα ura3-1 trp1-1 leu2-3,112 his3-11,15 can1-100 fzo1Δ::LEU2 mdm30Δ::KanMX6* pRS416-FZO1 | 2a-b, 2c, 5c, S2a-c | This study |
| *FZO1 ubp2Δ* (MCY654) | MCY572 | *MATα ura3-1 trp1-1 leu2-3,112 his3-11,15 can1-100 fzo1Δ::LEU2 ubp2Δ::HIS5* pRS416-FZO1 | 2a-b, 2c, 5c, S2a-c | This study |
| *FZO1 ubp2Δ mdm30Δ* (MCY694) | MCY572 | *MATα ura3-1 trp1-1 leu2-3,112 his3-11,15 can1-100 fzo1Δ::LEU2 ubp2Δ::HIS5 mdm30Δ::KanMX6* pRS416-FZO1 | 2a-b, 2c, 5c, S2a-c | This study |
| DF5 (MCY408) | DF5 | *Mata his3-Δ200 leu 2-3,2-112 lys2-801 trp1-1(am) ura 3-52* | 1c-d, 4c, 7a-d, 8a-d, S1a-c, S3a, S8a-b, S9a | 4 |
| DF5 (MCY 415) | DF5 | *Matα his3-Δ200 leu 2-3,2-112 lys2-801 trp1-1(am) ura 3-52* | 4c | 4 |
| *rsp5Δ+spt23** (MCY881) | DF5 | *Mata his3-Δ200 leu 2-3,2-112 lys2-801 trp1-1(am) ura 3-52 rsp5Δ::HIS3* pSPT23URA3 | 1c-d, 4c, S1a-c | This study |
| *rsp5Δ+spt23** (MCY882) | DF5 | *Matα his3-Δ200 leu 2-3,2-112 lys2-801 trp1-1(am) ura 3-52 rsp5Δ::HIS3* pSPT23URA3 | 4c | This study |
| *mdm30Δ* (MCY409) | DF5 | *Mata his3-Δ200 leu 2-3,2-112 lys2-801 trp1-1(am) ura 3-52 mdm30Δ::KanMX6* | S1a-b | This study |
| *rsp5Δ mdm30Δ* (MCY964) | MCY881 | *Mata his3-Δ200 leu 2-3,2-112 lys2-801 trp1-1(am) ura 3-52 mdm30Δ::KanMX6 rsp5Δ::HIS3* pSPT23URA3 | S1a-b | This study |
| *ubp2Δ* (MCY965) | DF5 | *Mata his3-Δ200 leu 2-3,2-112 lys2-801 trp1-1(am) ura 3-52 ubp2Δ::KanMX6* | 1c-d, S1c, S3a | This study |
| *rsp5Δ ubp2Δ* (MCY966) | DF5 | *Mata his3-Δ200 leu 2-3,2-112 lys2-801 trp1-1(am) ura 3-52 ubp2Δ::KanMX6 rsp5Δ::HIS3* pSPT23URA3 | 1c-d, S1c | This study |
| DF5 OM45-GFP (#779) | DF5 | *Mata OM45-GFP::KanMX4, his3-Δ200, leu2-3, lys2-801, trp1-1(am), ura3-52* | 5d, S6 | This study |

| | | | | |
|---|---|---|---|---|
| DF5 Mito-mCherry (#980) | DF5 | *Mata his3-Δ200, leu2-3,2-11::TEF-Mito-mCherry:LEU2, lys2-801, trp1-1, ura3-52* | 5d, S6 | This study |
| *UBP2-6HA* (MCY968) | W303 | *MATa ura3-1 trp1-1 leu2-3,112 his3-11,15 can1-100 UBP2-6HA::KanMX6* | 2d-e, 3e-f, 5b, S2d, S3c-d, S5b | This study |
| *UBP2-6HA mdm30Δ* (MCY1031) | MCY970 | *MATa ura3-1 trp1-1 leu2-3,112 his3-11,15 can1-100 UBP2-6HA::HIS3 mdm30Δ::KanMX6* | 3e-f, 5b, S3c-d | This study |
| *UBP2-6HA rup1Δ* (MCY1384) | MCY968 | *MATa ura3-1 trp1-1 leu2-3,112 his3-11,15 can1-100 UBP2-6HA::KanMX6 rup1Δ::TRP1* | S3d | This study |
| *MDM30* (MCY971) | W303 | *Mat α ura3-1 trp1-1 leu2-3,112 his3-11,15 can1-100 mdm30Δ::KanMX6 + pRS316-MDM30* | 3a-b, 3g, , 4a-b, 4f, 5a, 6a-c, S3b, S3f, S4a-c | This study |
| *MDM30 ubp2Δ* (MCY996) | MCY971 | *Matα ura3-1 trp1-1 leu2-3,112 his3-11,15 can1-100 mdm30Δ::KanMX6 ubp2Δ::LEU2 + pRS316-MDM30* | 3a-d, 4f, S3b, S3e-f, S4b, S5a | This study |
| *ubp2Δ* (MCY1251) | W303 | *MATα ura3-1 trp1-1 leu2-3,112 his3-11,15 can1-100 ubp2Δ::NATMX6* | 1a, S4b, S5b | This study |
| *ubp2Δ rup1Δ* (MCY1389) | MCY 1251 | *MATα ura3-1 trp1-1 leu2-3,112 his3-11,15 can1-100 ubp2Δ::NATMX6 rup1Δ::TRP1* | S3e | This study |
| *MDM30 OLE1-9MYC* (MCYO2) | MCY971 | *Matα ura3-1 trp1-1 leu2-3,112 his3-11,15 can1-100 mdm30Δ::KanMX6 OLE1-9MYC::HIS3 + pRS316-MDM30* | 4e | This study |
| *OLE1-13MYC* (MCY1126) | MCY971 | *Matα ura3-1 trp1-1 leu2-3,112 his3-11,15 can1-100 mdm30Δ::KanMX6 OLE1-13MYC::HIS3 + pRS316-MDM30* | S8a | This study |
| *OLE1-13MYC ubp2Δ* (MCY1032) | MCY971 | *Matα ura3-1 trp1-1 leu2-3,112 his3-11,15 can1-100 mdm30Δ::KanMX6 ubp2Δ::LEU2 OLE1-9MYC::HIS3 + pRS316-MDM30* | S8a | This study |
| *ubp2Δ* (MCY1147) | MCY572 /DF5 | *Matα his3-Δ200 leu 2-3,2-112 lys2-801 trp1-1(am) ura 3-52 can1-100 ubp2Δ::HIS5* | 7a-c, 8b-d, S8a-b, S9a | This study |
| *mga2Δ* (MCY1078) | W303 | *Matα ura3-1 trp1-1 leu2-3,112 his3-11,15 can1-100 mga2Δ::KanMX6* | 7a-c, 8a-d, S8a-b, S9a | This study |
| *ubp2Δ mga2Δ* (MCY1098) | MCY 1147/ 1078 | *Matα ura3-1 trp1-1 leu2-3,112 his3-11,15 can1-100 ubp2Δ::HIS5 mga2Δ::KanMX6* | 7a-c, 8b-e, S8a-b, S9a, S9c | This study |
| *MDM30* (MCY970) | W303 | *Mat a ura3-1 trp1-1 leu2-3,112 his3-11,15 can1-100 mdm30Δ::KanMX6 + pRS316-MDM30* | 5a, 6a-c | This study |

## Supplementary Table 2: Plasmids used in this study.

| **Name (Collection number)** | **Description** | **Occurrence in the study** | **Reference** |
|---|---|---|---|
| pRS314 (MC219) | CEN,*TRP1*, Amp | 6b-c, 8b, 8d-e, S9 | 5 |
| pRS316 (MC218) | CEN,*URA3*, Amp | 6b-c | 5 |
| pRS423 (MC86) | 2 micron, *HIS3*, Amp | 3g, S3a, S3d | |
| pRS425 (MC87) | 2 micron, *LEU2*, Amp | 4a-b, 4e, S4a, S4c | |
| pRS416-FZO1 (MC322) | CEN, *FZO1* promoter-*FZO1*, *URA3*, Amp | *FZO1* shuffling plasmid | 6 |
| pRS314-FZO1 (MC250) | CEN, *FZO1* promoter-*FZO1*, *TRP1*, Amp | 5d, 6c, 8b-e, S7, S9 | 7 |
| pRS314-FZO1 K398R (MC278) | CEN, *FZO1* promoter-*fzo1 K398R*, *TRP1*, Amp | 8e, S9c | This study |
| pRS414-TEF-FZO1-13MYC (MC227) | CEN, *TEF1* promoter-*FZO1-13MYC*, *TRP1*, Amp | 1c, 1f, 2b, 2c, S1a, S2a | This study |
| pRS414-TEF-FZO1-13MYC K398R (MC342) | CEN, *TEF1* promoter-*fzo1-13MYC K398R*, *TRP1*, Amp | 2c | This study |
| pRS416-TEF-FZO1-13MYC (MC206) | CEN, *TEF1* promoter-*FZO1-13MYC*, *URA3*, Amp | 1b, | This study |
| pRS414-FZO1-13MYC (MC333) | CEN, *FZO1* promoter-*FZO1-13MYC*, *TRP1*, Amp | 2a, 5c, S1b-c, S2c | This study |
| pRS316-MDM30 (MC331) | CEN, *MDM30* promoter-*MDM30*, *URA3*, Amp | 3a-b, 3g, 4a-b, 4e-f, 5a, 6a-b, S3b, S3d, S4a, S4c, S7 | This study |
| pTEF-MDM30-MYC | 2 micron, *TEF1* promoter-*MDM30-MYC*, *URA3*, Amp | 3c-d, S5a | 8 |
| pTEF-fbox-MYC | 2 micron, *TEF1* promoter-*mdm30-MYC fbox*, *URA3*, Amp | 3c-d, S3e | 8 |
| pRS423-UBP2-6HA (MC345) | 2 micron, *UBP2* promoter-*UBP2-6HA*, *HIS3*, Amp | 3c-d, 3g, S3a, S3e-f, S5a | This study |
| pRS423-UBP2-6HA C745S (MC347) | 2 micron, *UBP2* promoter-*ubp2-6HA C745S*, *HIS3*, Amp | S3a | This study |
| pRS315-RSP5(MC292) | CEN, *RSP5* promoter-*RSP5*, *LEU2*, Amp | 4a-b, 4e, S4a, S4c | This study |
| pYX232-mitoGFP (MC214) | 2 micron, *TPI* promoter-*Su9-GFP*, *TRP1*, Amp | 7c, 8c | 9 |
| pYeL1-mtGFP (MC337) | CEN, *GAL10* promoter-*mtGFP*, *LEU2*, Amp | 4c, 5a, 6a-c | This study |
| pYeL1-mtRFP (MC336) | CEN, *GAL10* promoter-*mtRFP*, *LEU2*, Amp | 4c, 5a, 6a-c | This study |
| pSPT23URA3 (MC338) | CEN, *SPT23* promoter, *SPT23 (1-686)*, *URA3*, Amp<br>Construct allowing survival of *rsp5Δ* strains | 1c-d, 4c | 4 |

## Supplementary Table 3: Primers for plasmids used in this study.

| Name (Collection number) | Name | 5'-3' sequences |
|---|---|---|
| pRS314-FZO1 K398R (MC278) et pRS414-TEF-FZO1-13MYC K398R (MC342) | P85 : K398R_F | tgacctgtccccagaaacatatagacgtgcagctga |
| | P86 : K398R_R | tcagctgcacgtctatatgtttctggggacaggtca |
| pRS414-TEF-FZO1-13MYC (MC227) et pRS416-TEF-FZO1-13MYC (MC206) | P53 : Fzo1+1_Sal_F | cctcccgtcgacatgtctgaaggaaaacaac |
| | P54 : Fzo1Myc_Sal_R | cctcccgtcgacggcgcgaattcactagtgattg |
| pRS414-FZO1-13MYC (MC333) | P188 : Inspromendo-F | agggaacaaaagctggagctcaaaggagtttgtgtcgtttttcaccagg |
| | P189 : Inspromendo-R | gataacttcttgagtgagctccacgacgataatttaatgccgtttaat |
| pRS423-UBP2-6HA (MC345) | P175 : SpeI-UBP2-F | cctcacactagtatctagacaccgctatcaag |
| | P176 : UPB2-NotI-R | ggagtgcggccgcttttttcagtctccgatttt |
| pRS423-UBP2-6HA C745S (MC347) | P198 : UBP2-C745S-F | ggcattaataatatcgggaacaccagttacctaaattctttattacaat |
| | P199 : UBP2-C745S-R | attgtaataaagaatttaggtaactggtgttcccgatattattaatgcc |

## Supplementary Table 4: Primers used in this study for construction of strains.

| Name | Name | 5'-3' sequences |
|---|---|---|
| *FZO1* (MCY572) | P17 : Fzo1_F | ctgatatcacggatagaggcaaaacggtaggctcatttaacgcagctgaagcttcgtacgc |
| | P18 : Fzo1_R | cattatgtatattgatttgaaaagacctcatatatttacaagaatatgcataggccactagtggatctg |
| *FZO1 mdm30Δ* (MCY585) | P62 : Mdm30_Forward | cctgaacaatttttcggtattagttactaaaaggctcacatataccagctgaagcttcgtacgc |
| | P63 : Mdm30_Reverse | ggtgtaatagaatgtgtcaggatgctacttttggaaacctccttaaatatgagcataggccactagtggatctg |
| *FZO1 ubp2Δ* (MCY654) et *ubp2Δ* (MCY965) et *rsp5Δ ubp2Δ* (MCY966) et *MDM30 ubp2Δ* (MCY996) et *ubp2Δ* (MCY1251) | P82 : UBP2-F | aattaaaaagaaagcttttgttcaaggttaagaaggtataaggaacagctgaagcttcgtacgc |
| | P83 : UBP2-R | ttatggcaatagtgacattttacataaactcttcattgactaagagcataggccactagtggatctg |
| DF5 OM45-GFP (#779) | P157 : OM45-S3 | aagaatggaatgataagggtgatggtaaattctggagctcgaaaaaggaccgtacgctgcaggtcgac |
| | P158 : OM45-S2 | tgtatatatgttatgcgggaaccaaccctttacaattagctatctaactaatcgatgaattcgagctcg |
| *UBP2-6HA* (MCY968) | P173 : S3-UBP2 | tcaaacaaggacaagaaggtgatattgagccattgaaaagaattctaaagcgtacgctgcaggtcgac |
| | P174 : S2-UBP2 | acttatggcaatagtgacattttacataaactcttcattgactaagactaatcgatgaattcgagctcg |
| *MDM30 OLE1-9MYC* (MCYO2) | P217 : OLE1-F2 | tagtaagagaggtgaaatctacgaaactggtaagttctttcggatccccgggttaattaa |
| | P218 : OLE1-R1 | tttttatggtagttgcagttttgttattgtaatgtgatacgaattcgagctcgtttaaac |
| *mga2Δ* (MCY1078) | P238 : MGA2_F | catttaaaggcacttattgaaggtcattttggcgaacagaacatttcgttcagctgaagcttcgtacgc |
| *ubp2Δ mga2Δ* (MCY1098) | P239 : MGA2_R | ctgtcttttcattatacacacatatatatatatatatacgtaaaaaagcagagcataggccactagtggatctg |

## *Supplementary References*


1. Surma, M.A. *et al.* A lipid E-MAP identifies Ubx2 as a critical regulator of lipid saturation and lipid bilayer stress. *Mol Cell* **51**, 519-530 (2013).
2. Lam, M.H. & Emili, A. Ubp2 regulates Rsp5 ubiquitination activity in vivo and in vitro. *PLoS ONE* **8**, e75372 (2013).
3. Kandasamy, P., Vemula, M., Oh, C.S., Chellappa, R. & Martin, C.E. Regulation of unsaturated fatty acid biosynthesis in Saccharomyces: the endoplasmic reticulum membrane protein, Mga2p, a transcription activator of the OLE1 gene, regulates the stability of the OLE1 mRNA through exosome-mediated mechanisms. *J Biol Chem* **279**, 36586-36592 (2004).
4. Hoppe, T. *et al.* Activation of a membrane-bound transcription factor by regulated ubiquitin/proteasome-dependent processing. *Cell* **102**, 577-586 (2000).
5. Sikorski, R.S. & Hieter, P. A system of shuttle vectors and yeast host strains designed for efficient manipulation of DNA in Saccharomyces cerevisiae. *Genetics* **122**, 19-27 (1989).
6. Griffin, E.E. & Chan, D.C. Domain interactions within Fzo1 oligomers are essential for mitochondrial fusion. *J Biol Chem* **281**, 16599-16606 (2006).
7. Cohen, M.M. *et al.* Sequential requirements for the GTPase domain of the mitofusin Fzo1 and the ubiquitin ligase SCFMdm30 in mitochondrial outer membrane fusion. *J Cell Sci* **124**, 1403-1410 (2011).
8. Cohen, M.M., Leboucher, G.P., Livnat-Levanon, N., Glickman, M.H. & Weissman, A.M. Ubiquitin-Proteasome-dependent Degradation of a Mitofusin, a Critical Regulator of Mitochondrial Fusion. *Mol Biol Cell* **19**, 2457-2464 (2008).
9. Westermann, B. & Neupert, W. Mitochondria-targeted green fluorescent proteins: convenient tools for the study of organelle biogenesis in Saccharomyces cerevisiae. *Yeast* **16**, 1421-1427 (2000).
